# Practical Notes on Diagnosis and Treatment

**Published:** 1913-06-21

**Authors:** 


### PRACTICAL NOTES ON DIAGNOSIS AND TREATMENT.

**Pruritus Vulvæ.**

A METHOD of treatment sometimes successful is to apply blisters over the last dorsal and first lumbar vertebræ.

**Leucocytosis.**

IN a febrile case with difficulty in the diagnosis the presence of a leucocytosis points to pneumonia and away from influenza or typhoid fever.

**Aspirin in Carcinoma.**

IN inoperable cases of cancer where there is pain a trial should be given to aspirin before having recourse to morphine. Fifteen grains two or three times daily may be given.

**Malignant Endocarditis.**

BACTERIOLOGICAL examination of the blood is often negative in this disease, and in these circumstances it is wise to use injections of both anti-streptococcic and anti-staphylococcic serum.

**Irreducible Hæmorrhoids.**

WHERE there is difficulty in pushing hæmorrhoids back within the bowel, and consequent danger of strangulation, the application of a solution of adrenalin (1 in 2,000 to 1 in 1,000) may prove of great value.

**Gastric Ulcer.**

ANY young woman who suffers from pain and vomiting after food unrelieved by careful dieting and free doses of bismuth subnitrate, ought to be regarded as, at least probably, the victim of gastric ulcer, and should be treated by rest in bed, etc.

**Cancer of the Stomach.**

IF a test meal shows HCl in good quantity this is against a diagnosis of cancer, while the absence of HCl is decidedly in favour of cancer. In 80 to 90 per cent. of cases of gastric cancer HCl is either absent or present in small quantity only.

**Chorea.**

IT should be remembered that chorea sometimes expresses itself, not in the form of muscular spasm and inco-ordination, but in the form of muscular weakness or paralysis. This may be unilateral and unless the point is borne in mind a mistake in diagnosis may readily occur.

**Skin Rashes in Septicæmia.**

PATCHES of an erythematous skin rash, together with high temperatures, may for a time be the only evidences of septicæmia. It may be difficult at first to distinguish such a condition from scarlet fever, and the position is not eased when, as sometimes happens, well-marked desquamation occurs.

**Bismuth Subnitrate.**

IT is a mistake to prescribe the salts of bismuth in mixture with mucilage of tragacanth or other suspending agent. They should be given in cachets or as a powder placed on the tongue; if it is desired to order a mixture the bismuth salt should be prescribed with compound infusion of orange and a "shake the bottle" label be placed on the bottle.

**Graves's Disease.**

AMONG the occasional symptoms in this disease are glycosuria, orthostatic albuminuria, and movable kidney.

**Sciatica.**

IN obstinate cases a method to be remembered is fixation by means of a long splint, so as to secure immobilisation both of the hip and the knee-joint.

**Professional Activities.**

ACCORDING to Sir William Osler, the happiest and most useful lot given to man is to become a vigorous, whole-souled, intelligent general practitioner.

**The Personal Factor.**

THE late Dr. Moxon used to say: "It is quite as important to know what kind of a patient the disease has got as to know what kind of disease the patient has got."

**Syphilis.**

THE treatment of syphilis does not end with mercury, nor even with 606. Fresh air and an outdoor life are probably as helpful in syphilis as they are in pulmonary tubercle.

**Goitre.**

A CERTAIN number of cases of simple goitre undoubtedly improve under thyroid extract; one grain two or three times daily is a suitable dose in which to commence the administration.

**The Ophthalmoscope.**

"No defect in medical education seems so persistent as ophthalmoscopic training. It will be so until the inspection of the normal disc . . . is taught with the stethoscope at the beginning of practical medicine."—*Sir William Gowers.*

**Facial Paralysis.**

AN aid in the restoration of muscular efficiency is to advise the patient to practise attempts at movement by grimacing before a mirror for five minutes or so, two or three times a day. This may be advised, not at the outset of the paralysis, but in the course of a few days.

**Pulse Tracings.**

THERE is one feature about sphygmograms which conclusively shows that the tracing has been badly taken; it is the existence of a straight line, or plateau, at the summit of the wave. This means that the needle of the instrument is not swinging freely and such tracings should be disregarded.

**Pericarditis.**

OCCURRING in the course of rheumatic fever, pericarditis hardly adds to the immediate gravity of the condition, but as a complication of chronic renal disease pericarditis is of very serious omen; the same is true of pleurisy, and it is to be noted that each of these complications may develop without any rise of temperature, and even without pain. They are due, presumably, to a toxic state of the blood.

